# Creating Stretchable Electronics from Dual Layer Flex-PCB for Soft Robotic Cardiac Mapping Catheters

**DOI:** 10.3390/mi14040884

**Published:** 2023-04-20

**Authors:** Abdellatif Ait Lahcen, Alexandre Caprio, Weihow Hsue, Cory Tschabrunn, Christopher Liu, Bobak Mosadegh, Simon Dunham

**Affiliations:** 1 Dalio Institute for Cardiovascular Imaging, Department of Radiology, Weill Cornell Medicine, New York, NY 10021, USA; 2 Department of Clinical Sciences, College of Veterinary Medicine, Cornell University, Ithaca, NY 14853, USA; 3 Electrophysiology Section, Cardiovascular Division, Hospital of the University of Pennsylvania, Philadelphia, PA 19104, USA; 4 Department of Cardiology, Weill Cornell Medicine, New York, NY 10021, USA

**Keywords:** flexible PCBs, laser postprocessing, soft robotics, stretchable electronics, cardiac arrhythmia mapping

## Abstract

The authors present in this study the development of a novel method for creating stretchable electronics from dual-layer flex printed circuit boards (flex-PCBs) as a platform for soft robotic sensor arrays (SRSAs) for cardiac voltage mapping applications. There is a crucial need for devices that utilize multiple sensors and provide high performance signal acquisition for cardiac mapping. Previously, our group demonstrated how single-layer flex-PCB can be postprocessed to create a stretchable electronic sensing array. In this work, a detailed fabrication process for creating a dual-layer multielectrode flex-PCB SRSA is presented, along with relevant parameters to achieve optimal postprocessing with a laser cutter. The dual-layer flex-PCB SRSA’s ability to acquire electrical signals is demonstrated both in vitro as well as in vivo on a Leporine cardiac surface. These SRSAs could be extended into full-chamber cardiac mapping catheter applications. Our results show a significant contribution towards the scalable use of dual-layer flex-PCB for stretchable electronics.

## 1. Introduction

Cardiac mapping is a procedure used to characterize abnormal electrical activity in patients with cardiac arrhythmia, allowing operators to identify and treat the source of the arrhythmia using cardiac ablation [1,2]. This can be performed using various techniques such as electroanatomic mapping, which uses a combination of electrical signals and imaging to create a detailed map of the heart’s electrical activity [3]. Once the location of the abnormal activity is identified, the patient can then be treated; a separate ablation catheter is inserted and energy is applied locally (e.g., radiofrequency, electroporation, cryothermal) to destroy the problematic tissue and restore a normal cardiac rhythm. Additionally, non-invasive methods such as electrocardiography, echocardiography, magnetic resonance imaging, computed tomography, and other imaging modalities can be used in conjunction with mapping to help localize the arrhythmia [4,5,6,7,8]. Minimally invasive diagnostic catheters with one or many sensors at the tip allow electrophysiologists to collect electrical measurements from the heart’s interior surface [9,10]. By articulating the catheter, the sensors are moved around the cardiac surface. Signal and the location of the catheter at the time of collection can be aggregated to provide a more a comprehensive picture of the electrical activity. Mapping the electrical activity of the heart is a critical component for the diagnosis and treatment of arrhythmia [2].

The use of stretchable electrodes arrays allows for more conformable mapping and for simultaneous acquisition of electrical signals [9,11,12,13]. This can lead to a more accurate and detailed map of cardiac arrhythmia. More broadly, a wide variety of demonstrations have shown the potential of utilizing structured 2D/3D electrodes to provide highly conformal sensing for a wide variety of biomedical applications [14,15,16,17,18,19,20].

Furthermore, the use of high precision laser-based cutting and/or micromachining techniques have been used to create single and multilayer electronics with 3D and/or conformable systems. Stuart et al. (2021) demonstrated personalized and digitally manufactured wireless biosensors for indefinite collection of high-fidelity biosignals. The researchers used laser engraving to pattern flexible circuit boards The resulting devices were found to have high sensitivity and accuracy for measuring physiological signals [14]. Ausra et al. (2022) created a fully implantable cardiac stimulation and recording devices. The researchers used a laser cutter to pattern flexible circuit boards and then assembled the devices into a 3D structure. The resulting device was found to have high accuracy for pacing and defibrillation in animal models [15]. Jang et al. (2022) developed graphene e-tattoos for unobtrusive ambulatory electrodermal activity sensing on the palm. The researchers used a laser cutter to pattern graphene and serpentine ribbons, resulting in a conformal and stretchable device that can measure electrodermal activity with high sensitivity and accuracy [17].

Another approach, based on thermal masking, allows for self-aligned removal of Kapton to eliminate Kapton from flex-PCB substrates, while preserving it underneath electrical traces. This has been demonstrated previously and allows for selective substrate removal without need for alignment and registration, for low cost, scalable creation of stretchable electrics [9,21]. However, prior demonstrations were limited to single layer flex-PCBs. There is a need for higher densities of electrodes to achieve higher resolution mapping. One way to achieve this is to extend this self-aligned laser based postprocessing technique to dual and multi-layer flexible printed circuit boards (flex-PCBs). The use of dual-layer flex-PCBs allows for increased electrode densities as well as the ability to route signals past one another utilizing vias. Prior demonstrations illustrate a laser-based postprocessing technique that utilizes thermal masking. This approach, when utilized in conjunction with serpentine electrical traces, can yield stretchable electronic configurations that can accommodate up to ~30% strain [9,21]. While this process has been successfully demonstrated in single layer flex-PCB designs, additional considerations arise when processing dual layer flex-PCBs.

Laser-based postprocessing of flex-PCBs is a versatile and precise method that can be used to convert inelastic flex-PCBs into stretchable electrode arrays [9]. The laser postprocessing cutting technique using a thermal masking approach is a promising method for producing stretchable electronics [21,22]. The technique involves using a laser to selectively remove insulation layers from a single-layer flex-PCB. This, in conjunction with the use of serpentine electrode designs, increases the stretchability of the electronics by allowing the metallic traces to expand and contract without being constrained by the insulation layers. However, because insulating layers are preserved under the metallic traces, when arrays are integrated with elastomeric substrates (e.g., silicone, polyurethane), the resulting structure is mechanically graded in its thickness, dramatically enhancing the stretchability of the resulting features. This approach has been demonstrated to be effective in our previous research, and it offers a scalable way to produce stretchable electronics [9]. This method can provide a cost-effective and efficient way to produce stretchable electronics for various applications such as wearable devices, medical implants, and robotics.

In this study, we have developed as a proof-of-concept a novel method for creating stretchable electronics from dual-layer flex-PCB with serpentine sensor array designs. These stretchable electronics are used as a platform for soft robotic sensor arrays (SRSA) for cardiac mapping. There is a crucial need for devices that can embed multiple sensors and provide better signal acquisition for cardiac mapping applications. In this study, a detailed fabrication process of a dual-layer multielectrode flex-PCB soft robotic sensor array (SRSA) has been demonstrated. As far as we know, the present work is the first study that reports the use of self-aligned laser-postprocessed in multi-layer flex-PCB materials. Furthermore, we demonstrate the potential of these arrays to be utilized for cardiac mapping, by showing the SRSA’s ability to acquire electrocardiograms both in vitro as well as in vivo on a leporine heart. 

## 2. Experimental Section

### 2.1. Materials and Instrumentation 

#### 2.1.1. Fabrication Stack up for the Soft Robotic Sensor Array Actuator

**Figure 1:**
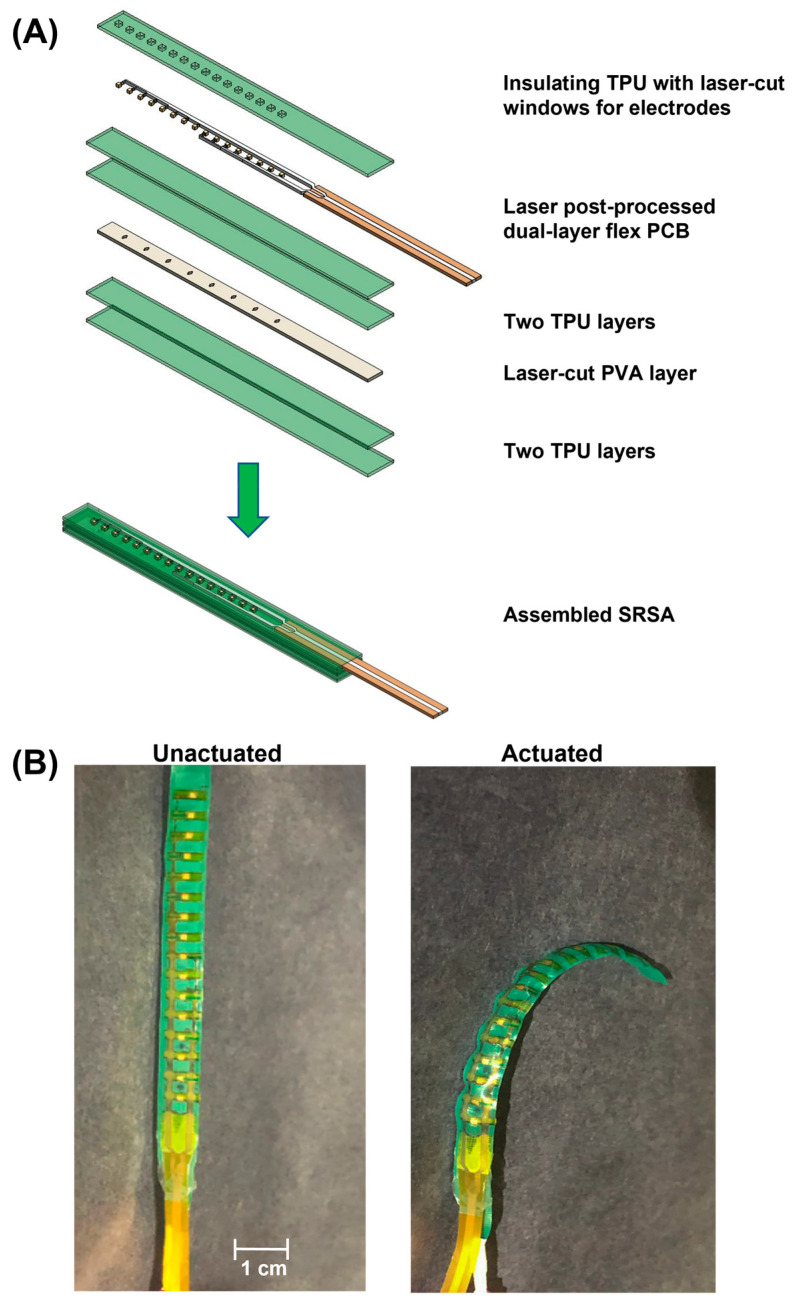
(**A**) Schematic image of layered stack up for the soft robotic sensor array (SRSA) actuator: thermoplastic polyurethane (TPU), flexible printed circuit board (flex-PCB), polyvinyl alcohol (PVA), and soft robotic sensing array (SRSA). (**B**) Real image of the laser postprocessed SRSA actuator before actuation and after actuation.

The stack-up of all the dual-layer flex-PCB SRSA layers used in this work are presented in Figure 1. The inelastic flex-PCB was designed and sourced from a conventional flex-PCB vendor (EPEC Engineered technologies, New Bedford, MA, USA). The fabrication stack up process allows for the integration of the flex-PCB electronics into an actuator. The components used are designed using Rhinoceros 3D software (Rhino 6, North Seattle, WA, USA). All the components used are prepared from sheets using a laser cutter (VL300 Universal laser systems, 30 W—600 × 300 mm) Universal laser systems, Scottsdale, Arizona, USA. This laser can cut and engrave a variety of materials up to 5 mm thick and engrave many materials including wood, plastics, metals, stone, and glass.

To fabricate the soft robotic sensor array (SRSA) actuator, several steps have been followed. As shown in Figure 1A, the PVA (polyvinyl alcohol) was laser cut into a suitable dimension to be sandwiched between the two layers of the green thermoplastic polyurethane. The purpose of using PVA is to avoid the bonding of the TPU layer and to serve as a sacrificial layer allowing the facilitation of the liquid flow during the hydraulic actuation [9]. As shown in Figure 1A, the flex-PCBs were laser postprocessed before integrating the electronics with the soft robotic actuators. On top of the electronics, an additional layer of TPU is heat-pressed to encapsulate them. This layer contains windows to allow the electrodes to be exposed during the data acquisition [9,22]. These different layers are assembled and heat pressed to induce bonding between polyurethane layers (Figure 1A). Once an actuator is complete, it is infused with water to dissolve the PVA layers creating a sealed hydraulic actuator. Figure 1B shows this actuator before and after actuation.

#### 2.1.2. In Vivo Voltage Mapping

The in vivo voltage mapping was studied using an open-surgical approach to acquire electrogram signals in leporine models. The electrical signals were acquired using a National Instrument data acquisition system controlled by a LabView homemade software (NI-6225, National Instruments Corporation, Austin, TX, USA). The leporine was anesthetized first and the chest was opened to expose the heart. The pericardial sac was stripped to allow access to the epicardial surface. Once the heart is exposed, the device is moved into contact with the cardiac surface to collect electrical signals. A 3D printed handle holder made from VeroClear material (Stratasys, Eden Prairie, MN, USA) with two slots allowed the actuators to expose only one specific electrode to the leporine heart surface. The handle also allows for irrigation with saline water to keep the heart surface moist, which helps to reduce friction, minimize damage to the cardiac tissue, and ensure the surface of the heart does not dry out. This not only enhances the safety of the procedure but also allows for more accurate and reliable results. These electrograms are postprocessed during and after the procedure to evaluate their quality. The leporine models remain under anesthesia for the duration of the recording and at the end of the recording.

## 3. Results and Discussion

### 3.1. Laser Postprocessing of Dual-Layer Flex-PCB Sensors

**Figure 2:**
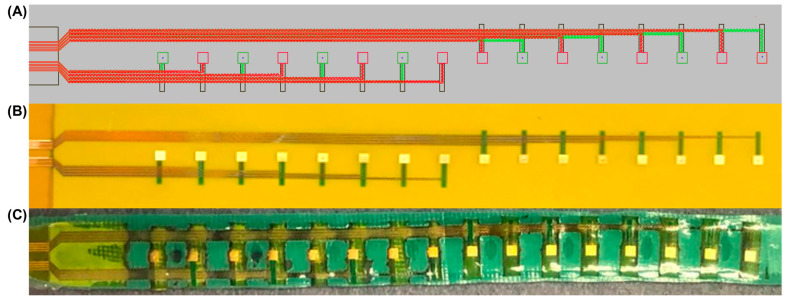
(**A**) The 2D CAD design of dual-layer flex-PCB, (**B**) real image of unprocessed dual-layer flex-PCB coated with an electroless nickel immersion gold (ENIG) finish, and (**C**) processed dual-layer flex-PCB ENIG actuator.

Figure 2 presents the dual-layer flex-PCB sensor arrays CAD design. As shown in Figure 2A, a 2D CAD design presents the dual-layer flex-PCB containing copper serpentine traces for both layers, an in between polyimide layer for insulation and 16 electrodes. In the regions where the density of electrical connects is defined by the limits of the flex-PCB fabrication, the dual layer design allows for twice the density of traces relative to a single layer design. Figure 2B shows the real image of the unprocessed flex-PCB. This type of actuator is made of a flex-PCB that has been coated with Electroless Nickel Immersion Gold (ENIG) finish, which provides a smooth and corrosion-resistant surface. Figure 2C shows the prepared laser postprocessed dual-layer flex-PCB actuator that will be used for the in vitro and in vivo voltage mapping experiments described in the sections below. The flex-PCBs are covered with a layer of green thermoplastic polyurethane (TPU) to provide insulation and allow the actuation of the flex-PCB.

**Figure 3:**
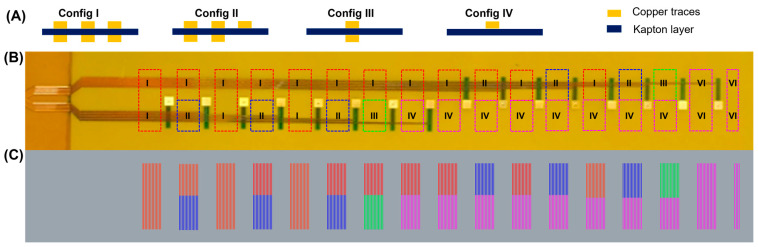
Schematic of various zones for dual-layer flex-PCB. (**A**) Schematics of four possible flex-PCB configurations: (I) multiple serpentines on both the top and bottom layers, (II) multiple serpentines on both top and bottom layers, plus a single layer serpentine on either layer, (III) single serpentine on both the top and bottom layers, and (VI) single serpentine on both either the top or bottom layer. (**B**) Image of dual-layer flex-PCB. (**C**) Image of laser cutting path with color-coded zones. Laser power values: (I) Red 11%, (II) Blue 8%, (III) Green 10%, and (IV) Magenta 8%. Laser speed: 20%.

The laser postprocessing of the flex-PCB stretchable electronics is essential for actuator functionality; unprocessed flex-PCB is too inextensible to accommodate the deformations associated with actuation. In this context, we have developed a laser-based postprocessing that allows for scalable fabrication of stretchable sensor arrays (Figure 3A). The dual-layer flex-PCBs were subjected to ultrasonication for 20 min and subsequently underwent a meticulous visual inspection to guarantee the complete removal of Kapton from between traces. Additionally, specialized equipment such as a microscope or a camera with high magnification is used to inspect the board’s surface. Any remaining Kapton is carefully removed using appropriate tools such as a pair of tweezers, taking care not to damage the underlying traces or components. It is worth noting that we successfully eliminated the Kapton between the copper traces in all areas except for configuration II, where the removal was only partial.

Figure 3 shows a schematic of various zones for dual-layer flex-PCB. We have marked the different laser-cutting paths with color-coded zones. As described in prior work, the power required to optimally remove the polyimide, while preserving it below the traces, depends on the density of traces which conduct heat away from the laser spot and reduce the temperature of the heating zone [21]. It is important to note that the dual-layer flex-PCB used in this study has different regions that require different laser power values than those needed for processing single-layer flex-PCBs. To address this, we have divided the postprocessed regions into four different configurations based on their design and complexity. As can be seen in Figure 3, these configurations include multiple serpentines on both top and bottom layers (Config. I), a combination of multiple serpentines and single-layer serpentines on either layer (Config. II), single serpentines on both top and bottom layers (Config. III), and single serpentine on both either the top or bottom layer (Config. IV). Table 1 provides a summary of the different scenarios for the laser postprocessing of the dual-layer flex-PCB ENIG. However, we acknowledge that in the case of configuration II, where single-layer and dual-layer traces are situated close to each other, the dual-layer sections could not be optimally postprocessed without potentially damaging the single-sided traces. This is because dual-layer traces are more efficient in transmitting heat from the laser spot and require higher powers to ensure complete removal of Kapton material from between the traces. 

It should be noted that using designs that do not include single sided traces would mitigate this limitation. Additionally, single sided traces could be incorporated such that they require the same conditions to postprocess as dual layer traces, by adding a “dummy” bottom layer trace that follows the same path as the top layer trace but does not connect electrically to any electrodes. All values of laser postprocessing presented in this study are optimized (Figures S1 and S2 in Supplementary Materials). Moreover, this laser postprocessing allows the preservation of the polyimide material underneath the copper traces resulting in a mechanically graded structure (in the z-axis), which improved durability relative to similar traces without intermediate stiffness supports. This demonstration shows the potential to utilize this method with multilayer flex-PCB, while achieving the same self-aligned material removal. This provides the potential to utilize this method to scalably convert circuits to stretchable arrays with more complex circuits designs that utilize overlapping traces and vias. The preservation of polyimide material and the use of laser post-processing method results in a high-quality flex-PCB that is durable, robust, and capable of meeting the demands of various electronic applications.

We have developed a detailed methodology for laser postprocessing of the dual-layer flex-PCB ENIG. We have identified different configurations for the postprocessing of different regions of the dual-layer flex-PCB and have acknowledged the limitations of this approach in certain scenarios.

One potential advantage of self-aligned laser-postprocessed dual-layer flex-PCBs is the ability to create stretchable electronics that can acquire signals from multiple sensors. This is particularly useful for cardiac mapping applications, where multiple sensors are needed to accurately map the electrical activity of the heart. Additionally, the use of dual-layer flex-PCBs allows for greater flexibility and stretchability. Furthermore, the precise alignment also allows for the creation of multielectrode arrays that can capture a larger area of electrical activity in the heart. Beyond this, the use of multilayer designs that incorporate vias allow for more advanced circuits where traces can overlap on different layers. Thus, this demonstration provides broader complexity. This methodology could be useful for researchers and engineers working on the development of flexible and stretchable electronics.

### 3.2. In Vitro Voltage Mapping Evaluations of SRSA

The as-prepared SRSA actuator’s capability to perform voltage mapping has been assessed in saline water. Indeed, the in vitro voltage acquisition was carried out using a single array of sensors that contains 16 electrodes of the flex-PCB ENIG actuator.

**Figure 4:**
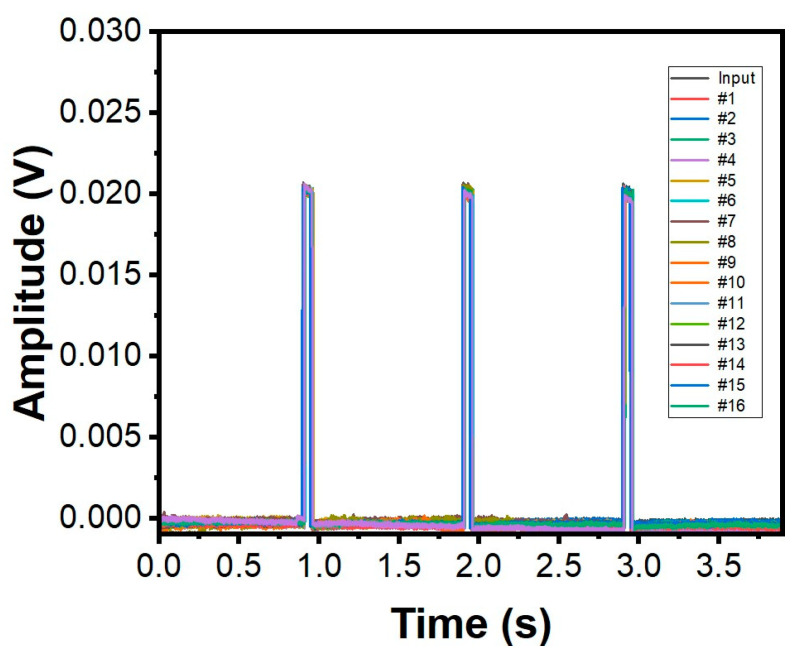
Input waveforms and measured waveforms using all the 16 electrodes on the SRSA in saline water. The input waveform has a voltage of 20 mV and a pulse width of 50 ms. # refers to the electrode number.

The in vitro study of the developed SRSA actuator was conducted to evaluate their performance in comparison to baseline measurements from an oscilloscope. The study involved taking simultaneous measurements using the oscilloscope at a frequency of 1 Hz, an input amplitude of 20 mV, and a pulse width of 50 ms. The choice of these experimental parameters is comparable to that of a realistic electrogram. The results of the study were represented in Figure 4, which shows the sensors data for low voltage and low pulse width in standard saline solution. All 16 electrodes in the SRSA exhibit similar response for the input voltage as shown in Figure 4. The data demonstrates the SRSA actuator’s ability to effectively read electrograms, indicating their potential for use in further research.

### 3.3. Validation of Electronic Readings In Vivo Measurements

**Figure 5:**
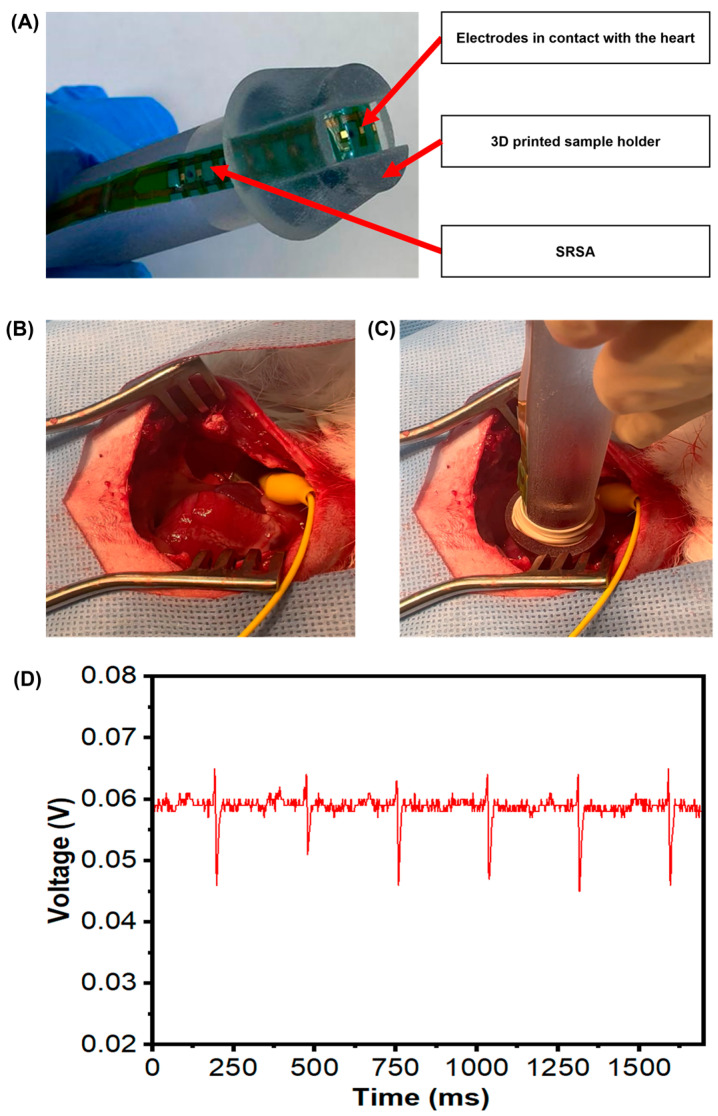
In vivo validation of electronic readings (**A**) flex-PCB SRSA mounted on a 3D printed sample holder. (**B**) Exposed leporine heart before collecting signals. (**C**) SRSA mounted on a 3D printed holder in contact with leporine heart. (**D**) Acquired signal using flex-PCB ENIG with a 500 Hz sampling rate.

In this study, leporine animal models are used to perform the in vivo measurements using a smaller version of our device and/or only a subset of the sensors on the larger device. The single flex-PCB SRSA was mounted on a 3D printed sample holder electronic readings; we have used a single linear array of the dual flex-PCB ENIG actuator as shown in Figure 5A to be used in contact with the leporine heart surface. 

To achieve this goal, we characterized signal acquisition from the epicardial surface of an exposed leporine heart. As described in the experimental section, an open surgical approach has been used to gain access to the epicardial surface to acquire electrogram signals (Figure 5B). Once the heart is visualized, the SRSA mounted on the 3D printed handle holder is properly positioned to collect the electrical signals from the surface of the heart (Figure 5C). The data acquisition system started the recording of the electrogram signals using the flex-PCB ENIG device under a 500 Hz sampling rate (Figure 5D). The outcome of these experiments successfully confirmed the proper acquisition of electrogram signals from the leporine heart with high accuracy.

### 3.4. Discussion

Soft robotic features with embedded sensing have been shown to exhibit high levels of conformability to complex tissue and anatomy. In this study, we have demonstrated a new method for creating stretchable electronics using dual-layer flex-PCBs allowing for scalable fabrication of flexible circuits with higher electrode densities. Here, greater electrode density allows for more detailed mapping of electrical signals in the heart. Below we detail a process to produce a dual-layer multielectrode flex-PCB using laser based postprocessing and show their integration into soft robotic sensor array (SRSA) using. The fabrication process for this SRSA is described in detail, including the design of the electrode pattern, the selection of materials, and the laser postprocessing of the flex-PCBs. We have also provided relevant parameters necessary to achieve optimal postprocessing of the flex-PCBs with a laser cutter. We demonstrate the ability of the dual-layer flex-PCB SRSA to acquire electrical signals both in vitro and in vivo on a leporine cardiac surface. This result suggests the potential for these SRSAs to be utilized for full-chamber cardiac mapping catheter applications.

This study represents an advancement towards the scalable use of dual-layer flex-PCBs for stretchable electronics. It provides the ability to scalably fabricate stretchable sensor designs with higher density. The use of dual-layer flex-PCBs as a platform for stretchable electronics provides a versatile and scalable solution for creating electronics that can conform to complex surfaces and detect electrical signals from multiple sensors simultaneously. Although this study has demonstrated the feasibility of the proposed approach, it also shows the broader potential to construct multilayer circuits with vias and interconnects to allow stretchable designs from a scalable components [23]. However, there are several challenges that need to be addressed for practical applications. For example, the long-term stability and reliability of the dual-layer flex-PCB SRSA need to be evaluated, and the compatibility of the materials with biological tissues needs to be further investigated. However, because they are constructed from medical grade polyurethanes and PCB materials, they are likely to demonstrate similar results. Our results from this study provide a promising direction for the development of stretchable electronics for biomedical applications. The proposed approach could lead to the development of new medical devices that can improve patient outcomes by providing accurate and high-performance signal acquisition for cardiac mapping and other biomedical applications.

## 4. Conclusions

To sum up, this research paper demonstrates a promising method for the fabrication of stretchable electronics using dual-layer flex-PCBs and laser postprocessing technique. The resulting stretchable SRSA actuators exhibited excellent performance in acquiring electrical signals in vitro and were further validated using a leporine model to assess the acquisition of electrograms from a real cardiac surface. Our experiments confirmed that proper voltage mapping signals could be acquired with high accuracy, demonstrating the potential of these stretchable electronics for use in various medical applications. These approaches should be applicable to larger multiactuator cardiac mapping system. This represents a meaningful demonstration of a highly conformable device design, showing the benefits that enhanced conformability provide. To conclude, our findings suggest that the use of dual-layer flex-PCBs and laser postprocessing could provide a promising approach for the fabrication of stretchable electronics with a wide range of potential applications in medical diagnostics and treatment.

## Figures and Tables

**Table 1:** Optimized laser postprocessing parameters for all zones for dual-layer flex-PCB.

Configuration Type	Laser Post-Processing/Optimized Conditions	Microscope Top View Image	Microscope Bottom View Image
I	Multiple serpentines on both the top and bottom layers Red 11%	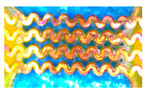	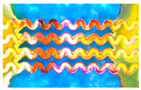
II	Multiple serpentines on both top and bottom layers, plus a single layer serpentine on either layer Blue 8%	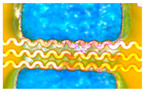	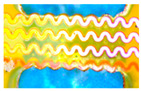
III	Single serpentine on both the top and bottom layer Green 10%	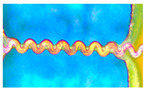	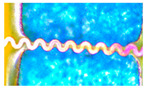
IV	Single serpentine on both either the top or bottom layer Magenta 8%	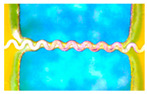	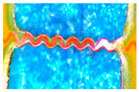

## Data Availability

All data needed to evaluate the conclusions of this manuscript are present in the paper and/or the Supplementary Materials. Additional data related to this paper may be requested from the authors.

## Supplementary Materials

The following supporting information can be downloaded at: https://www.mdpi.com/article/10.3390/mi14040884/s1, Figure S1: Image of dual-layer Flex-PCB before and after laser postprocessing at optimized power values. Laser speed: 20% title; Figure S2: Microscope images for the different laser postprocessed flex-PCB regions under different laser power values.
