# Supplementary material for: Creating Stretchable Electronics from Dual Layer Flex-PCB for Soft Robotic Cardiac Mapping Catheters
Source: Micromachines (Basel). 2023 Apr 20;14(4):884. doi: 10.3390/mi14040884 (PMC10146302; doi:10.3390/mi14040884)
Supplement: Supplementary file 1 [file micromachines-14-00884-s001.zip › micromachines-2320045-supplementary.pdf]

Creating Stretchable Electronics from Dual Layer Flex-PCB for Soft Robotic Cardiac Mapping Catheters

Abdellatif Ait Lahcen [1], Alexandre Caprio [1], Weihow Hsue [2], Cory Tschabrunn [3], Christopher Liu [4], Bobak Mosadegh [1,*] and Simon Dunham [1,*]

[1] Dalio Institute for Cardiovascular Imaging, Department of Radiology, Weill Cornell Medicine, New York, NY 10021, USA
[2] Department of Clinical Sciences, College of Veterinary Medicine, Cornell University, Ithaca, NY 14853, USA
[3] Electrophysiology Section, Cardiovascular Division, Hospital of the University of Pennsylvania, Philadelphia, PA 19104, USA
[4] Department of Cardiology, Weill Cornell Medicine, New York, NY 10021, USA
* Correspondence: bom2008@med.cornell.edu (B.M.); sid2012@med.cornell.edu (S.D.)

<u>Supporting materials</u>

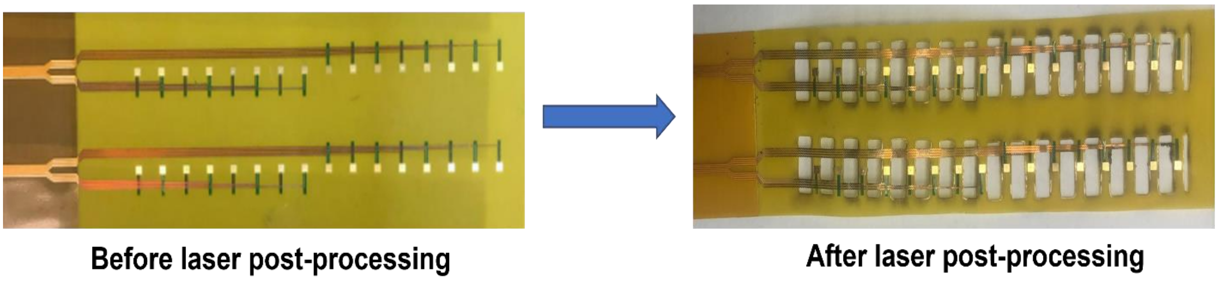

Figure S1. Image of dual-layer Flex-PCB before and after laser postprocessing at optimized power values. Laser speed: 20%.

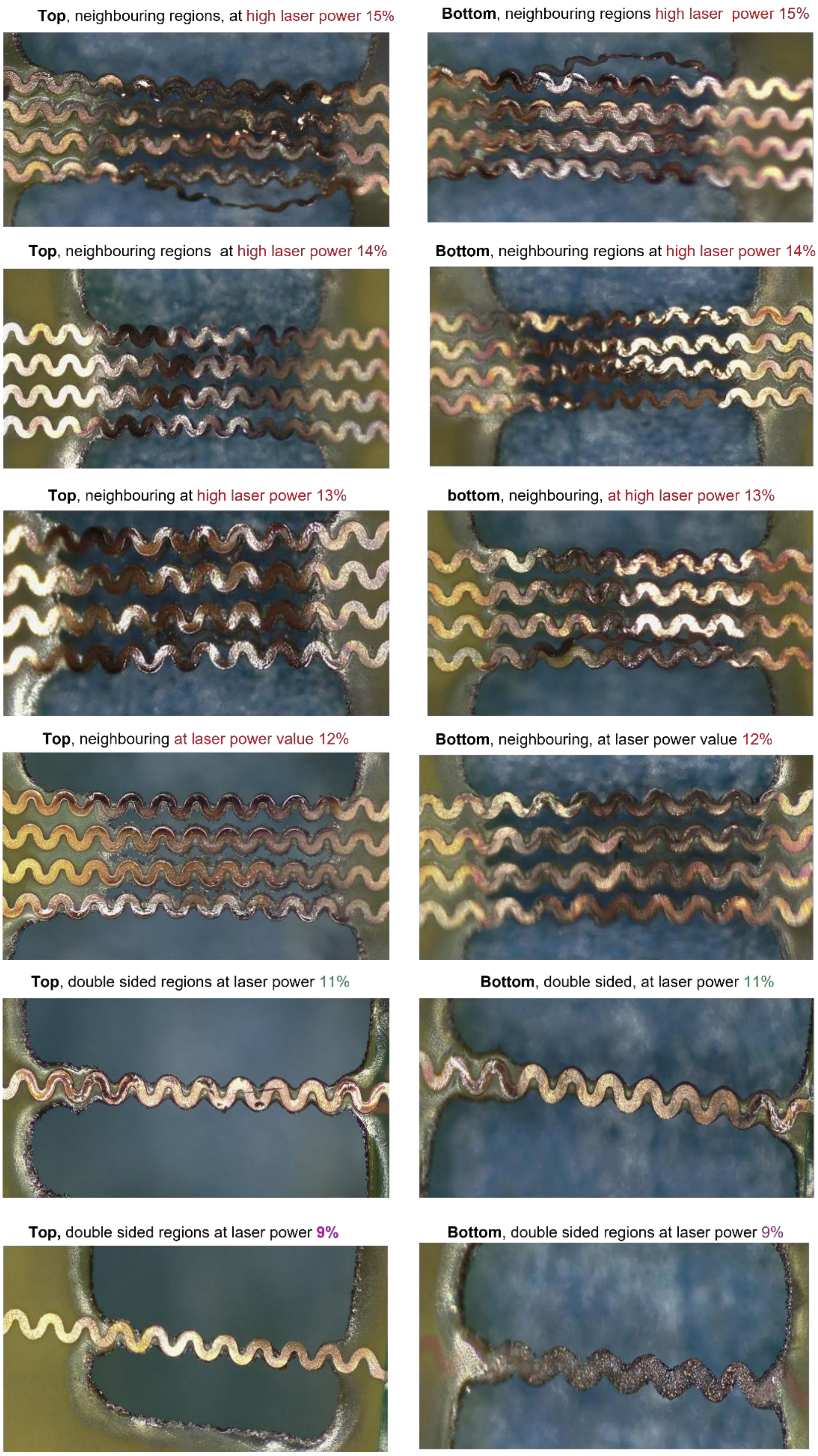

Figure S2. Microscope images for the different laser postprocessed flex-PCB regions under different laser power values.
